# Balneotherapy for osteoarthritis: a systematic review

**DOI:** 10.1007/s00296-023-05358-7

**Published:** 2023-06-10

**Authors:** Carmela Protano, Mario Fontana, Andrea De Giorgi, Daniela Marotta, Nicholas Cocomello, Serena Crucianelli, Angela Del Cimmuto, Matteo Vitali

**Affiliations:** 1 Department of Public Health and Infectious Diseases, Sapienza University of Rome, 00185 Rome, Italy; 2 Department of Biochemical Sciences, Sapienza University of Rome, 00185 Rome, Italy; 3 Department of Clinical Internal, Anesthesiological and Cardiovascular Sciences, Sapienza University of Rome, 00185 Rome, Italy

**Keywords:** Osteoarthritis, Balneology, Mineral water, Systematic review as topic

## Abstract

This systematic review is aimed to evaluate the effects of balneotherapy with thermal mineral water for managing the symptoms and signs of osteoarthritis located at any anatomical site. The systematic review was conducted according to the PRISMA Statement. The following databases were consulted: PubMed, Scopus, Web of Science, Cochrane Library, DOAJ and PEDro. We included clinical trials evaluating the effects of balneotherapy as a treatment for patients with osteoarthritis, published in English and Italian language, led on human subjects. The protocol was registered in PROSPERO. Overall, 17 studies have been included in the review. All of these studies were performed on adults or elderly patients suffering from osteoarthritis localized to knees, hips, hands or lumbar spine. The treatment assessed was always the balneotherapy with thermal mineral water. The outcomes evaluated were pain, palpation/pressure sensibility, articular tenderness, functional ability, quality of life, mobility, deambulation, ability to climb stairs, medical objective and patients’ subjective evaluation, superoxide dismutase enzyme activity, serum levels of interleukin-2 receptors. The results of all the included studies agree and demonstrated an improvement of all the symptoms and signs investigated. In particular, pain and quality of life were the main symptoms evaluated and both improved after the treatment with thermal water in all the studies included in the review. These effects can be attributed to physical and chemical-physical properties of thermal mineral water used. However, the quality of many studies resulted not so high due and, consequently, it is necessary to perform new clinical trial in this field using more correct methods for conducting the study and for processing statistical data.

## Introduction

By definition, osteoarthritis results from complex mechanical, biological, molecular and enzymatic interactions, all finally leading to the articular tissue deterioration and affecting the whole joint [1]. Osteoarthritis is considered one of the most common musculoskeletal diseases and one of the ten most disabling diseases in developed countries; it is estimated that, worldwide, 7% of the population (more than 500 million people) are affected by osteoarthritis, with a much higher risk for women. The number of people affected by this condition increased by 48% from 1990 to 2019, and in 2019 osteoarthritis was the 15th leading cause of years lived with disability [2].

In the United States, osteoarthritis afflicts more than 32.5 millions of adults, and it is considered one of the most expensive disease in term of treatment [3]. Indeed, in 2013, osteoarthritis was reported as the second most expensive sanitary condition among those treated in US hospitals, determining a total expenditure of 16.5 billion dollars, which represents the 4.3% of all hospitalization costs [4]. In Italy, the situation does not differ from other countries: osteoarthritis represents, alone, the 72.6% of the musculoskeletal system diseases. The rate of osteoarthritis is directly proportional to age, especially when involving individuals ranging from 75 to 79 years of age; men are more affected than women up to the fifth decade of life, whereas, after menopause the trend is inverted with the latter having a higher prevalence [5].

Osteoarthritis can affect any moving joint but some specific forms of the disease have a selective localization as gonarthrosis (also called knee osteoarthritis), which is the osteoarthritis affecting one or both knees, which globally represents the most frequent localization, with a prevalence of 3.8% [6]. The therapy of the osteoarthritis includes conservative approaches (pharmacological and non-pharmacological) and/or surgical ones. The target of therapy consists in relieving the pain, maintaining the articular mobility and maximizing the articular and global function. Main non-pharmacological conservative treatments consists of some physical measures as rehabilitation, support devices, exercises to increase muscles strength, flexibility and endurance, yoga patient’s education through daily life suggestions and thermal therapy, which includes massages, hydrotherapy, mud therapy and balneotherapy [7–10]. Several studies compared balneotherapy with other osteoarthritis standard treatments, revealing that balneotherapy has greater long-term effects on life quality in people with severe disability, decreasing pain sensation and articular stiffness and increasing mobility of the limbs [11]. Balneotherapy, due to its several beneficial effects, has been recommended by The OARSI guidelines (Osteoarthritis Research Society International) as a complementary non-pharmacological treatment for gonarthrosis, coxarthrosis and polyarticular osteoarthritis [12]*.*

Besides, balneotherapy is cited for its positive effects on osteoarthritis also in the EULAR (European League Against Rheumatism) recommendations for the health professional’s approach to pain management in inflammatory arthritis and osteoarthritis [13], while ARC (American College of Rheumatology/Arthritis Foundation) made a conditionally recommendation for thermal intervention [14]. A recent systematic review and meta-analysis evaluated the effects of balneotherapy and spa therapy on knee osteoarthritis, but just focalizing the attention on the on quality of life of patients [15]. The present systematic review is aimed at evaluating the effects of balneotherapy as a treatment to manage symptoms and signs of osteoarthritis, considering all the possible anatomical sites, in adults and elderly.

## Methods

### Research strategy

This systematic review was performed by PRISMA conceptual framework (Preferred Reporting Items for Systematic Reviews and Meta-Analyses) Statement [16]. The following bibliographic and citations databases were consulted: PubMed (Medline), Scopus, Web of Science (Science and Social Science Citation Index), Cochrane Library, PEDro (Physiotherapy Evidence Database) and DOAJ (Directory of Open Access Journals). The research was performed using predefined key words and MeSH terms and relying on Boolean operators AND–OR through the following string: “balneotherapy AND osteoarthritis”. The research considered all papers from each database consulted up to the 20th April 2023. The protocol was registered on the PROSPERO platform (reference number: CRD42021258598).

### Study selection

In this review we included all the original papers aimed at evaluating the effects of thermal water for balneotherapy as a treatment for the management of symptoms and signs of osteoarthritis, published in English and Italian language and performed on humans. We considered eligible observational studies, semi-sperimental and sperimental studies, while we excluded case reports, case series, letters to editors, commentaries, editorials and other papers reporting studies without new objective data. References of critical and systematic reviews and/or meta-analyses were assessed to find additional useful papers. No time limits were set. PICO model was used for structuring the research question, as follows:

*Population* Patients older than 18 years having osteoarthritis.

*Intervention* Balneotherapy.

*Control* Bathing in drinking water or procedures other than balneotherapy.

*Outcomes* Improvement of symptoms and signs of osteoarthritis.

The exclusion criteria led to reject all reports not fulfilling both the requirements of this review and the predetermined inclusion criteria.

All the references found by bibliographic research were transferred on Zotero software (Center for History and New Media (CHNM) at George Mason University (GMU)) for duplication removal and for assessing the relevance of each paper. As a first step, four investigators (M.V., A.D.G., D.M., C.P.) independently verified the agreement to selection criteria of any potential eligible study by reading title and abstract. Subsequently, all the papers considered potentially relevant were red in full-text. Any possible disagreement about the paper selection was solved through open-minded discussion.

### Main outcome variables

We considered all the possible improvements in signs and symptoms of knee osteoarthritis during and after balneotherapy treatment evaluated in the included articles.

### Risk of bias assessment

At the end of the review process, all the full-texts considered eligible were controlled clinical trials with the exception to one which not including a control group [17].

To date, no universally accepted checklist to evaluate the methodological quality of clinical trials testing non-pharmacological therapeutic treatment is available. Thereby, following the results of some systematic reviews on non-pharmacological treatment [18–20] or concerning the specific area investigated in the present review [21, 22], we evaluated the risk of bias of each included study using the CLEAR NPT checklist (Checklist to Evaluate a Report of a Non pharmacological Trial), specifically elaborated for non-pharmacological clinical trials by an expert group through the Delphi method [23]. Moreover, we used also the checklist elaborated by Forestier et al. [22] in a recent systematic review conducted to assess the effects of spa therapies on knee osteoarthritis.

Four investigators (M.V., A.D.G., D.M., C.P.) independently assessed the quality of each study using both the checklists described above. Disagreements were solved by discussion between the investigators. As already established by other authors [22], a score between 10 and 8 represents a low bias risk, a score ranging between 7 e 5 stands for a medium risk and a score lower than 5 has to be considered a high bias risk. The evaluation of the quality of the study without the control group was performed according to a previous similar systematic review [24]: considering the scientific value of the journal, the sample size and the methods used for patients’ assessment. However, due of the lack of a control group, the bias risk was considered high.

## Results

**Fig. 1:**
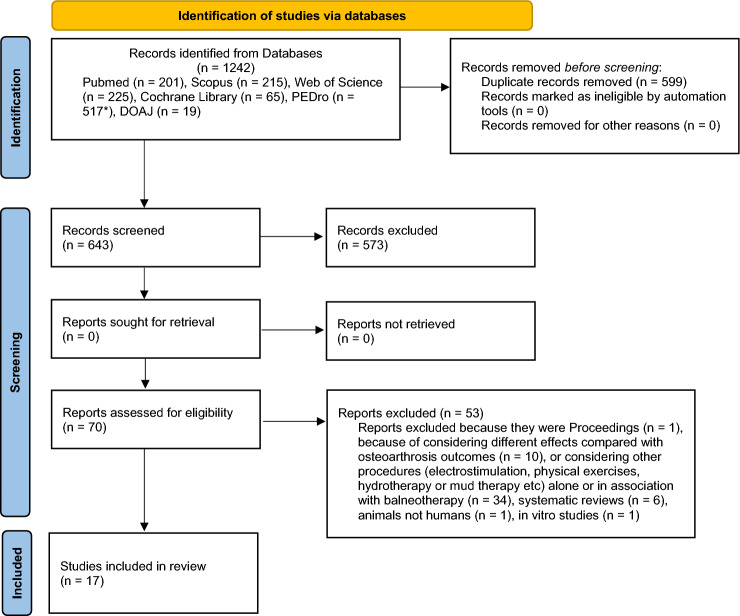
Flow chart describing the research strategy

Figure 1 shows the details of the review process. At the end of the selection process, 17 studies have been included in the review. The first research performed on PubMed, Scopus, Web of Science, Cochrane Library, PEDro and DOAJ databases produced 1242 bibliographic citations of which, after the duplicate removal 599 left; 573 were further excluded for not fulfilling the inclusion criteria by reading title and abstract.

The remaining 70 reports were examined in detail to exclude any other paper not respecting the inclusion criteria. From this ulterior selection, additional 53 reports were rejected because they were proceedings, or because considering different effects than the osteoarthritis outcomes, or because considering other treatments (electrostimulation, physical exercise, hydrotherapy or mud therapy, etc.) alone or associated to balneotherapy. Some other reports were excluded for being systematic reviews or studies performed on animals or in vitro rather than on humans. The references of these reports were assessed to find any possible additional relevant citations, but none of them satisfied the previously described inclusion criteria.

For each report included in this systematic review the following data were summarized: essential bibliographic data, thermal center and belonging country, source of funding, study design, osteoarthritis localization, population/patients’ features as gender and age, description of the main balneotherapy treatment and the control one, final results and original concluding remarks, bias risk. All these data are presented in Table 1.

**Table 1:** Features of the studies included in the review

Study; Thermal center, Country Study design Source of funding Osteoarthritis localization	Sample size and population main features	Balneotherapy treatment	Control/other groups procedure	Results and original concluding remarks of authors	Drugs used during treatments	Quality control using CLEAR NTP checklist
Szucs et al. [25]; Puspokladany, Hungary Double blinded, controlled clinical trial Not reported Knee	62 (number of procedures and controls not reported); 40–70 years old	20 min of bathing in thermal water for 3 consecutive weeks except Sundays	20 min of bathing in drinking water having the same temperature of thermal water for 3 consecutive weeks except Sundays	Significant reduction of motion pain and pressure sensibility or articular tenderness in subjects having bath in thermal water by the end of the treatment period compared to controls	Standard doses of pain-killer or non-steroid type anti-inflammatory drugs	4 yes, 6 not reported High bias risk
Nguyen et al. [26]; Vichy, France Randomized controlled clinical trial Private Lumbar spine, knee and hip	188 (91 procedures and 97 controls); 71 F (procedures); 82 F (controls); average age: 64 ± 7 (procedures); 63 ± 6 (controls)	20 min of bathing in thermal water for 3 consecutive weeks	Routine outpatient care at home for 3 consecutive weeks	Significant reduction of pain and significant improvement of functional ability and quality of life at 4 and 24 weeks after treatment respect to before balneotherapy treatment compared to the control group	Analgesic or non-steroidal anti-infiammatory drugs	3 yes, 3 no, 4 not reported High bias risk
Ekmekcioglu et al. [27]; Kurzentrum Baden bei Wien, Austria Randomized, single blinded for investigators, controlled clinical trial Not reported Not reported	38 (19 procedures and 19 controls); 11 F (procedures); 13 F (controls); average age: 52 ± 3 (procedures); 47 ± 4 (controls)	20 min of bathing in thermal water for 3 consecutive weeks + spa therapies	The same spa therapies without balneotherapy for 3 consecutive weeks	Significant reduction of the superoxide dismutase enzyme activity by the end of balneotherapy and compared to before the start of treatment	Not reported	4 yes, 2 no, 4 not reported High bias risk
Kovács and Bender [28]; Cserkeszölö, Hungary Randomized, double blinded, controlled clinical trial Not reported Knee	58 (31 procedures and 27 controls); other features not reported	30 min/day for 15 consecutive days of knee bathing in thermal water	30 min/day for 15 consecutive days of knee bathing in “placebo” drinking water (mixed with thermal water) having the same temperature of thermal water	Significant reduction of pain and palpation sensibility, significant improvement of mobility, deambulation, ability to climb stairs, outcomes of medical objective assessment and subjective assessment of patients right after the treatment and after 3 months in patients receiving balneotherapy compared to before the start of treatment	No drug therapy allowed	5 yes, 1 no, 4 not reported Medium bias risk
Odabaşi et al. [29]; Sandikli, Turkey Single blinded for investigators, controlled clinical trial Not reported Knee	49 (25 procedures and 24 procedures + mud therapy); 18 F (procedures); 17 F (procedures + mud therapy); average age: 60,4 ± 6,1 (procedures); 60,3 ± 6,2 (procedures + mud therapy)	20 min twice a day of bathing in thermal water for 8 consecutive days	20 min, once a day for 8 consecutive days of bathing in thermal water + mud therapy with thermal mud	Even if therapy combining thermal bath and mud applications seems to have greater improvement than the thermal bath alone, this difference may be not relevant from a clinical point of view. Both therapy regiments determine significant reduction of pain and significant improvement of disability and functional ability (according to physical exercises) at the end of treatments compared to before the start	No drug therapy allowed	3 yes, 2 no, 2 not reported 2 no for double blind, but similar concomitant treatments Medium bias risk
Yurtkuran et al. [30]; thermal center not reported, Turkey Randomized, double blinded, controlled clinical trial Not reported Knee	52 (27 procedures and 25 controls); 25 F (procedures); 24 F (controls); average age: 52.9 ± 6.8 (procedures); 55.5 ± 6.2 (controls)	20 min, once a day, 5 days/ week for a total of 2 weeks bathing in thermal water	20 min, once a day, 5 days/week for 2 consecutive weeks of bathing in drinking having the same temperature of thermal water	Significant reduction of pain and significant improvement of functional ability and quality of life at 2 and 12 weeks compared to before the start of treatment and significant reduction of pain and articular tenderness at 2 and 12 weeks since balneotherapy compared to control group	No drug therapy allowed	4 yes, 1 no, 5 not reported High bias risk
Alp et al. [31]; thermal center not reported, Turkey Randomized, double blinded, controlled clinical trial Not reported Knee	47 (23 procedures and 24 controls); 1 F (procedures); 1 F (controls); average age: 54.34 ± 6.4 (procedures) 56 ± 4.32 (controls)	20 min, 1 time per day, 5 days per week for 2 weeks of dive in thermal water	20 min, 1 time per day, 5 days per week for 2 consecutive weeks of bathing in drinking water having the same temperature of thermal water	Significant reduction of pain and serum levels of interleukin-2 receptors at 2 and 12 weeks compared to before the start of balneotherapy. Significant reduction of pain during motion at 2 weeks and serum levels of interleukin-2 receptors at 2 and 12 weeks since balneotherapy compared to control group	No drug therapy allowed	7 yes, 3 not reported Medium bias risk
Bálint et al. [32]; Nagybaracska, Hungary Randomized, double blinded, controlled clinical trial Not reported Both of knees	52 (27 procedures and 25 controls); 17 F (procedures); 16 F (controls); age ranging from 50 to 75	30 min, once a day, 5 days/ week for 4 weeks of bathing in thermal water	30 min, once a day, 5 days/ week for 4 weeks of bathing in drinking water having the same temperature of thermal water	Significant reduction of pain due to balneotherapy compared to the control group lasting for 3 months after the end of treatment with thermal water	No drug therapy allowed	5 yes, 1 no, 3 not reported, 1 no for single blind for therapists, but similar concomitant treatments and withdrawal and loss rate at follow-up Medium bias risk
Evcik et al. [33]; thermal center not reported, Turkey Controlled clinical trial Not reported Knee	80 (25 procedures, 29 controls and 26 other controls); 23 F (procedures) 22 F (controls) 24 F (other controls); average age: 55 ± 8.7 (procedures), 57.4 ± 9 (controls) 59.6 ± 9.2 (other controls)	20 min, once a day, 5 days/ week for 2 weeks of bathing in thermal water	Local application of mud therapy and hot packs, at 42 °C per 20 min, once a day, 5 days/week for 2 consecutive weeks	Significant reduction of pain and significant improvement of functional ability and quality of life at 2 and 3 months compared to before the start and to the control group	If needed patients were allowed to take paracetamol in a maximum dose of 1500 mg per day	2 yes, 2 no, 6 not reported High bias risk
Kiliçoğlu et al. [17]; Balikesir, Turkey Non controlled clinical trial Not reported Knee	30; 15 F; age ranging from 49 to 77	20 min, twice a day, for 2 consecutive weeks except Sundays of bathing in thermal water	Not performed	Significant improvement of function (checked by physical exercises), pain and quality of life, concomitant disability reduction at 2 weeks of since balneotherapy compared to before treatment start	Not reported	Good scientific value of the journal, feasible included sample size and methods. High bias risk due to lack of a control group
Fioravanti et al. [34]; Siena, Italy Randomized, single blinded, controlled clinical trial Not reported Knee	60 (30 procedures, 30 controls); 12 F (procedures), 18 F (controls); average age: 69.33 ± 7.63 (procedures), 72.45 ± 7.14 (controls)	20 min, once a day, 6 days/ week for 2 consecutive weeks of bathing in thermal water	Routine outpatient care at home	Significant reduction of pain and significant improvement of functional ability and quality of life at 2 weeks and 3 months compared to before the start of balneotherapy and to control group	Patients were advised to continue their established pharmacological treatments, with the exception of analgesic drugs (acetaminophen) and NSAIDs (Diclofenac, Piroxicam, Naproxen, Aceclofenac), which were to be noted daily in a diary. No corticosteroid, hyaluronic acid infiltrations or chondroprotective agents allowed	6 yes, 2 no, but similar concomitant treatments and withdrawal and loss rate at follow-up, 2 not reported Low bias risk
Horváth et al. [35]; Gunaras, Hungary Randomized, single blinded, controlled clinical trial No funding Hands	63 (21 procedures at 36 °C, 21 procedures at 38 °C, 21 controls); 17 F (procedures at 36 °C), 16 F (procedures at 38 °C), 18 F (controls); average age: 63.5 ± 4.7 procedures at 36 °C; 62.3 ± 4.8 procedures at 36 °C; 63.8 ± 4.4 controls	20 min, 5 days/week for 3 consecutive weeks of bathing in thermal mineral water at 36 °C + 20 min of magnetotherapy, 5 days/week for 3 consecutive weeks of bathing in thermal mineral water at 38 °C + magnetotherapy	Only magnetotherapy	Significant reduction of pain and significant improvement of functional ability during the follow-up due to balneotherapy compared to control group. No differences reported with different water temperatures	No drug therapy allowed	6 yes, 2 no, 2 not reported Medium bias risk
Kovács et al. [36]; Mezőkövesd, Hungary Randomized, double blinded, controlled clinical trial Not reported Hands	45 (24 procedures; 21 controls); 23 F (procedures), 19 F (controls); age ranging from 47 to 73 (47–71 procedures; 50–73 controls)	20 min, 5 days/week for 3 consecutive weeks of bathing in thermal water	20 min, 5 days/ week for 3 consecutive weeks of bathing in drinking water having the same temperature of thermal water	Significant reduction of pain and disability, significant quality of life improvement right after balneotherapy compared to before and to control group. Significant reduction of pain and disability at 6 months after treatment compared to before. Significant reduction of pain and disability at 3 months compared to the control group	No non-steroid drugs allowed. They were allowed to take paracetamol or metamisole if necessary	5 yes, 1 no, 4 not reported Medium bias risk
Kulisch et al. [37]; Lago di Hévíz, Hungary Randomized, single blinded, controlled clinical trial Not reported Knee	77 (38 procedures, 39 controls); 30 F (procedures), 30 F (controls); average age: 65.6 ± 6.4 (procedures), 65.7 ± 7.7 (controls)	20 min, 5 days/ week for 3 consecutive weeks of bathing in thermal water	20 min, 5 days/week for 3 consecutive weeks of bathing in drinking water having the same temperature of thermal water	Significant reduction of pain and significant improvement of functional ability and quality of life at 3 and 15 weeks compared to before the start and to control group	Nonsteroidal anti-inflammatory and chondroprotective therapy	8 yes, 2 no, but similar concomitant treatments and withdrawal and loss rate at follow-up, 2 not reported Low bias risk
Şahin-Onat et al. [38]; thermal center not reported, Turkey Controlled clinical trial No funding Knee	46 (19 procedures, 27 controls); 13 F (procedures), 23 F (controls); average age: 71.0 ± 8.2 (procedures), 70.6 ± 7.3 (controls)	20 min, 5 days/week for 3 consecutive weeks of bathing in thermal water at 38 °C + physiotherapy	Only physiotherapy	Significant reduction of pain and disability and significant improvement of walking speed right after balneotherapy compared to before and to control group	No drug therapy allowed	1 yes, 3 no, 6 not reported High bias risk
Kovács et al. [39]; Mezőkövesd, Hungary Randomized, single blinded, controlled clinical trial Not reported Hip	41 (21 procedures and 20 controls); average age: 59.14 ± 7.55 procedures and 60.66 ± 7.6 controls	20 min, 5 days/week for 3 consecutive weeks of bathing in thermal water + physical exercises at home	Only physical exercises at home	Significant reduction of stiffness by the end of balneotherapy compared to the control group. Significant reduction of pain and stiffness, improvement of functional ability and quality of life after 12 weeks of balneotherapy compared to control group	Not reported	7 yes, 1 no, 2 not reported Medium bias risk
Hanzel et al. [40]; Szigetvár, Hungary Randomized, double blinded, controlled clinical trial Not reported Knee and/or Hip	50 (26 procedures and 24 controls); 17 F (procedures) and 16 F (controls); average age: 66.22 ± 4.68 procedures and 67.43 ± 4.95 controls	30 min, 5 days/week for 3 consecutive weeks of bathing in a jacuzzi filled with thermal water	30 min, 5 days/week for 3 consecutive weeks of bathing in a jacuzzi filled with tap water	Significant reduction of pain and improvement of function and quality of life right after the balneotherapy treatment and at 3 months compared to before the start and to control group	Not reported	6 yes, 1 no, 3 not reported Medium bias risk

Each of the 17 articles described a clinical trial, of which 16 controlled [25–40] and 1 designed without control group [17]. 8 studies were performed in Hungary, 6 studies in Turkey and 3 respectively in France, Austria and Italy. The time of publication ranged from 1989 [25] to 2018 [40]. All the subjects considered in these trials were adults or elderly patients affected by osteoarthritis localized in various anatomical sites, such as knees, hips, hands or lumbar spine.

Participants to some clinical trials [25, 26, 33, 34, 36, 37] continued to take some medications during the study, while others from other trials [17, 27, 39, 40] were not allowed to take any drugs. The remaining studies [28–32, 35, 37, 38] did not report specifics regarding the use of drugs during the trials.

The proposed treatment was balneotherapy, consisting of 20 or 30 min/day of bathing, once or twice a day, for 2 to 3 weeks of total duration, coupled or not to other spa therapies, as magnetotherapy or physiotherapy only when homogeneously performed on both “sperimental” and “control” group.

The outcomes considered were expressed in term of pain, palpation/pressure sensibility, articular tenderness, functional ability, quality of life, mobility, deambulation, ability to climb stairs, medical objective and patient’s subjective evaluation, superoxide dismutase enzyme activity, serum levels of interleukin-2 receptors. These outcomes were assessed at the end of the treatment and/or after a 3 months’ follow-up period, compared to those presented before the treatment by the study group and/or by the control group. Some parameters as pain, the quality of life and functional ability have been determined by specific and validated scores, while others as the functional ability, sensibility to pressure or palpation and articular tenderness were determined by specific exercises or handlings. Serum levels of superoxide dismutase enzyme and interleukin-2 receptors have been determined in laboratory. All the study demonstrated an improvement in the outcomes assessed following the balneotherapy treatment respect to those detected before the treatment and/or those of the control group. In particular, the main results of all the reviewed studies evidenced an improvement of patient’s conditions after balneotherapy in terms of:

1. significant reduction of pain (16/17 studies);
2. significant reduction of pressure/palpation sensibility (2/17);
3. significant reduction of articular tenderness (2/17);
4. significant improvement of functional ability, mobility, deambulation, ability to climb stairs and reduction of disability (2/17);
5. significant improvement of quality of life (8/17);
6. significant improvement according to the objective medical assessment (1/17);
7. significant improvement according to the subjective patient’s assessment (1/17);
8. significant reduction of superoxide dismutase enzyme activity (1/17)
9. significant reduction of serum levels of ilterleukin-2 receptors (1/17).

According to the scores obtained applying the checklist CLEAR NTP, seven studies (including the non controlled study) showed a high bias risk, eight studies presented a medium bias risk and two studies a low bias risk. Furthermore, the quality assessment related to external and statistical validity of each included study highlighted some improvable methodological aspects. However, the matching outcomes reported by all the studies analyzed in this review attenuate the intrinsic relevance of bias, consolidating the whole scientific evidence. All the studies showed a significant improvement of one or more specific osteoarthritis signs and/or symptoms.

## Discussion

The most relevant finding of this review is that all the studies included demonstrated that balneotherapy improves signs and symptoms of osteoarthritis affecting different joints, increase the superoxide dismutase enzyme activity and the serum levels of interleukin-2 receptors. This result is in line with those reported by previous reviews [15, 24, 41]. In particular, a Cochrane review performed in 2008 [41] recovered silver-level evidence about the beneficial effects of baths in thermal mineral water compared to no treatment, but highlighted that the scientific evidence produced until the period of the review was weak due to the poor methodological quality and to the lack of an appropriate statistical analysis and data presentation. The two more recent reviews in this field found that balneotherapy can significantly improve the quality of life of patients with osteoarthritis [15, 24] and reduce pain, non-steroidal anti-inflammatory drug use, and functional limitation [24]. However, both these reviews were conducted considering just knee osteoarthritis. We considered all the possible anatomical sites affected by this condition.

Beneficial effects associated to the balneotherapy are due both to physical and chemical properties of thermal mineral water used for this treatment. Indeed, balneotherapy with thermal water exerts clinically useful effects through a double mechanism: the first is associated to the use of water (hydrotherapy) and it is determined by mechanical influences and hydrostatic pressure. The second one is related to the use of a specific type of mineral water (crenotherapy), and it depends on the chemical and physical–chemical properties of the water [42–45]. First of all, the high temperature of the thermal mineral waters used for the treatments of osteoarthritis exerts an analgesic action and influences muscle tone, promoting the relaxation of striated and smooth muscles. In particular, the effects of heat are characterized by an increase in the temperature of the skin, subcutaneous tissue and muscles, with a decrease in muscle tone [46]. High water temperature also influence the sensation of pain and induces sedation and muscle relaxation and increases mobility. Besides, immersing the body in mineral water allows for easier mobilization of the joints and facilitates muscle strengthening. In addition, hyperemia in periarticular sites (capsules, ligaments, tendon insertions), determined by heat stimulation, can contribute to the removal of inflammatory cytokines and chemokines, reducing pain. Finally, high temperature determines a series of neuroendocrine reactions, stimulating the hypothalamic–pituitary–adrenal axis with the consequent release of adrenocorticotropic hormone and cortisol, well-known for their anti-edema and anti-inflammatory action [47, 48].

Another important factor contributing to the to the improvement of symptoms and signs of osteoarthritis is the presence, in thermal mineral water of sulfur both in the reduced form of hydrogen sulfide (also called hydrogen sulfide) and in the more oxidized form of sulfate ion. In a systematic review made by Cheleschi et al. [50], all the scientific evidences of in vitro studies were reported, and the results highlighted that sulfurous thermal mineral waters have several beneficial effects, in particular on the antioxidant system. In fact, oxygen and nitrous free radicals production by polymorphonuclear leukocytes decreases after incubation of these cells in sulfurous thermal mineral water. Specifically, this water has a chondroprotective effect on chondrocytes and cartilage. In vitro, chondrocytes incubation with sulfurous water inhibits the release of nitrous oxide, E2 prostaglandins, metalloproteinases, TNF-α, Interleukine-6 and Interleukine-8, all molecules having a crucial role in the inflammatory processes and cartilage degradation in osteoarthritis [49].

The present systematic review has some limitations. First of all, we did not carry out statistical analysis of the results of the studies included in the review because we considered all anatomical sites potentially affected by osteoarthritis and, thus, we did not compare possible changes in symptoms and signs in different sites. Besides, each of the included studies used different methodological approaches for conducting the trial and for assessing changes in symptoms and signs of osteoarthritis after the balneotherapy treatment. Besides, symptoms and signs of osteoarthritis are highly variable and this could cause heterogeneity in the results. Moreover, some symptoms and signs (pain or quality of life) are evaluated in many studies, while for other parameters, there is not enough number of articles to draw strong evidence. However, the present systematic review is the first describing a picture of the effects of balneotherapy in symptoms and signs of osteoarthritis affecting all the anatomical sites. Notice that participants to some clinical trials continued to take some medications during the study, generating a possible confounding on the real effect of balneotherapy on the improvement of symptoms and signs of osteoarthritis. Another limitation of the present review is related to the quality of the studies included: fifteen studies showed a medium or high bias risk, while just two studies presented a low bias risk. Nevertheless, the results of all the studies agree and demonstrated a significant improvement of one or more specific osteoarthritis signs and/or symptoms after the balneotherapy treatments.

In conclusion, the main finding of the present systematic review is that all the included studies found a significant improvement in signs and/or symptoms of osteoarthritis, whatever the anatomical location of the disease. In particular, pain and quality of life were the main symptoms evaluated, and both improved after the treatment with thermal water in all the studies included in the review. For other parameters, the number of articles is not enough to draw strong evidence. However, the quality of the studies included are at medium or high risk of bias in many cases due to the not completely appropriate methodological approaches used to carry out the study and to elaborate the results. Consequently, it is necessary to perform new clinical trials using more correct methods for conducting the clinical trials, more advances techniques for processing statistical data and considering also those signs and symptoms still little studied.

## Data Availability

The data can be requested from the corresponding author.
